# Design of Complex Solid‐Solution Electrocatalysts by Correlating Configuration, Adsorption Energy Distribution Patterns, and Activity Curves

**DOI:** 10.1002/anie.201914666

**Published:** 2020-02-11

**Authors:** Tobias Löffler, Alan Savan, Hajo Meyer, Michael Meischein, Valerie Strotkötter, Alfred Ludwig, Wolfgang Schuhmann

**Affiliations:** ^1^ Analytical Chemistry—Center for Electrochemical Sciences (CES) Faculty of Chemistry and Biochemistry Ruhr University Bochum Universitätsstrasse 150 44780 Bochum Germany; ^2^ Chair for Materials Discovery and Interfaces, Institute for Materials Faculty of Mechanical Engineering Ruhr University Bochum Universitätsstrasse 150 44780 Bochum Germany

**Keywords:** catalyst screening, electrocatalysis, high-entropy alloys, intrinsic activity, oxygen reduction reaction (ORR)

## Abstract

Complex solid‐solution electrocatalysts (also referred to as high‐entropy alloy) are gaining increasing interest owing to their promising properties which were only recently discovered. With the capability of forming complex single‐phase solid solutions from five or more constituents, they offer unique capabilities of fine‐tuning adsorption energies. However, the elemental complexity within the crystal structure and its effect on electrocatalytic properties is poorly understood. We discuss how addition or replacement of elements affect the adsorption energy distribution pattern and how this impacts the shape and activity of catalytic response curves. We highlight the implications of these conceptual findings on improved screening of new catalyst configurations and illustrate this strategy based on the discovery and experimental evaluation of several highly active complex solid solution nanoparticle catalysts for the oxygen reduction reaction in alkaline media.

## Graphical abstract

**Elementary**: The effect of changing elements within high‐entropy electrocatalysts with respect to changes in adsorption energies and the correlated characteristic activity curves is analyzed. This concept is verified for the oxygen reduction reaction (ORR) in alkaline media. A targeted design of complex solid‐solution electrocatalysts is significantly enhanced by only screening equiatomic alloys without the need to scan the whole composition range.

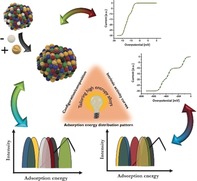

## Introduction

Despite remarkable achievements in designing novel electrocatalysts,^1^ state‐of‐the‐art catalysts are still often based on noble metals. This is especially important for complex multi electron–proton transfer reactions, such as the oxygen reduction reaction (ORR) and the oxygen evolution reaction (OER), in which scaling relations play an important role and novel approaches are required for a breakthrough.^2^ High entropy alloys (HEAs) forming a complex solid solution (CSS) in the form of nanoparticles (NPs) have recently been discovered as a new class of electrocatalysts.^3^ We propose the use of the term CSS instead of HEA when referring to electrocatalysis, since the additional refinement of atomic arrangement is the foundation of the unprecedented properties, as not all “HEAs” are indeed stabilized by high entropy. The unique interaction of many neighboring elements directly next to each other is the basis of an immense possible number of different active sites, which cover a wide range of adsorption energies.^4^ Optimization of the composition allows for increasing the number of active sites with favorable adsorption energy for the reaction of interest, and thus, the optimal choice of elements and their respective ratio can generate very active catalysts as long as a CSS phase is formed. The successful use of CSSs as electrocatalysts for several reactions, such as ammonia synthesis,^5^ CO oxidation,^6^ CO_2_ reduction,^7^ the ORR,^6^, ^8^ hydrogen evolution reaction (HER),^9^, ^10^, ^11^ or the OER^9^, ^11^, ^12^ was shown confirming universal applicability. However, the complex underlying working principles are still widely unknown with a lack of a conceptual framework. Hence, in‐depth understanding is required to tailor new catalysts for specific reactions. Adjusting of the activity of ORR catalysts was shown experimentally by tailoring the CSS composition,^8^ a strategy which could be rationalized by a theoretical study of the adsorption energy distribution pattern (AEDP)^4^ and its implication on the specific electrochemical behavior.^3^ However, it was not yet shown which effect different AEDPs have on activity curves and which effects are expected for addition or replacement of elements within the CSS phase.

Herein, we elucidate these fundamental concepts and discuss which information about the AEDP can be gained simply by analyzing the experimental activity curves. We highlight how this can significantly enhance the discovery of active catalysts and show experimental results for ORR catalysts where these concepts are tested. Thus, we reveal intrinsically active CSS catalysts, which can be further optimized for their implementation in catalyst films.

## Results and Discussion

### Deriving the Shape of Intrinsic Activity Curves

**Scheme 1:**
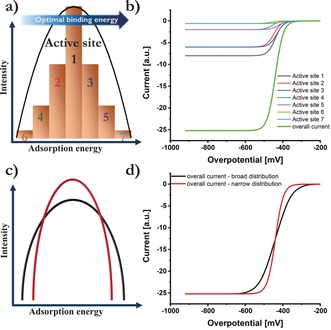
a) Simplified scheme to illustrate active site distribution within one adsorption peak. Active sites of similar activity are grouped together and summarized as one site, whose intensity depends on the amount of similar sites. b) Visualized intrinsic current response in the kinetic region of these grouped active sites, considering their activity as well as the intensity. For symmetrical distribution, the overall current response representing one peak still follows a consistent exponential increase, followed by a plateau current once active site limitation is reached. The activity is governed by the position of the peak maximum regarding optimal binding energies. c) Visualization of two different scenarios of a narrow and a broad distribution of an adsorption peak. d) Respective change of the “slope” in the overall current response as highlighted in (b) with regard of the peak distribution shown in (c). Whereas the inflection point is not affected, a reversed deviation at lower or higher currents is obtained. An exponential increase as predicted by the Butler–Volmer equation is still preserved for symmetric peak shapes.

The random arrangement of five or more elements in a single‐phase solid solution yields a continuous and unique AEDP, which was recently theoretically derived by Batchelor et al.^4^ Considering on‐top adsorption of reaction intermediates, there are as many adsorption peaks as there are elements in the CSS, with all active site motifs with one specific element in the active site center forming one of those peaks. When two‐ or three atoms form the active sites, the number of possible centers and thus the number of adsorption peaks increases even more. To postulate the measured electrochemical response of such a distribution pattern, we simplified the model by reducing the number of active sites within one adsorption peak to 7, while preserving the shape of the distribution pattern (Scheme 1 a). In this hypothetical example, the optimal binding energy is at higher binding energies relative to the peak, implying that active site 7 has the highest activity, followed by active site 5 until finally active site 6 follows with the least favorable binding energy. Plotting the kinetic activity curves while considering that active site 1 yields the highest current (since it represents the highest number of active sites in the original distribution pattern), seven individual curves are obtained, which sum up to the overall measured curve (Scheme 1 b). For a symmetrical peak shape, all deviations from average activity cancel out and a consistent exponentially increasing overall curve is obtained, even though it consists of a vast number of active site contributions of different activity. For irregular peak shapes, the absence of a counteracting active site of same intensity, but opposite distance to the average position induces a marginal distortion, which slightly affects the linearity in Tafel plots even if the kinetic region is analyzed. Additionally, the peak distribution, whether it is narrow or broad, affects the “slope” of the polarization curve in the same way as the transfer coefficient in the Butler–Volmer equation does (Scheme 1 c, d). Accordingly, Tafel analysis should be reconsidered for CSS catalysts and its interpretation extended to include variations in peak distribution. It should be noted that the inflection point is not affected.

Since there are multiple adsorption peaks for CSSs, each will contribute one exponentially increasing current curve of different activity depending on the position of the peak maximum relative to the optimal binding energy. Hence, in principle the number of “current waves” adding up equals the number of elements in the CSS, with the intensity affected by the molar ratio and the activity governed by the relative position of the respective peak maximum regarding optimal binding energies. Mass‐transport effects may limit the visibility of lower active “current waves”. For reactions with rather low mass‐transport limits, such as the ORR, it gets more likely to only see the most active wave. However, when the composition is not optimized and the distance and intensity of the two or even three most active peaks regarding optimal binding energies is rather similar, the combined contributions can be observed.

### Effect of Configuration Alteration

The effect of adding or replacing elements within the CSS phase is schematically depicted in Scheme 1, based on the previously discussed AEDP‐activity curve correlation. For an elemental catalyst, only one fixed and distinct binding energy exists if only the most active of the few facets such as (111) is considered,^13^ which yields an exponentially increasing activity curve based on the position relative to the optimal binding energy. At higher overpotentials, it transitions to a plateau either due to active‐site limitation or more likely due to mass‐transport limitation yielding a plateau current for hemispherical diffusion towards single NPs.^14^ For CSSs, catalytic curves can attain many shapes since the distances between the current wave segments are directly correlated to the distances of the peak maxima within the AEDP. In this scheme, only equiatomic compositions are considered and the active‐site‐limited plateau currents for each wave segment are set to the same value. For optimized compositions, the altered peak intensities have a direct influence on the current intensities of the respective waves, that is, a well‐pronounced peak close to the optimal binding energy will yield a wave segment at low overpotentials with high currents, which represents the ideal result for optimization of CSS catalysts. However, the complexity of the interaction between neighboring elements prevents simple predictions of the AEDP and hence the activity. One would need to either calculate the AEDP for each configuration (which elements constitute the CSS) with consecutive optimization of composition (molar ratio of elements in a CSS) and/or measure each catalyst individually in a screening process. Since each change in composition impacts activity, screening of the whole composition range would be required with the related challenge of time and effort. However, modulation in composition mainly affects the intensity with only minor shifts in adsorption energy of each peak.^4^ Thus, one important conclusion of the concepts discussed is the possibility to significantly reduce the effort of a screening process by evaluating equiatomic alloys and assess the intrinsic activity of the most active current wave. By this approach, promising configurations can be identified and the number of catalysts, which have to be experimentally investigated, can be significantly reduced. In a second step, optimization of the composition can be performed, now also including the current magnitude of the most active wave segment.

**Scheme 2:**
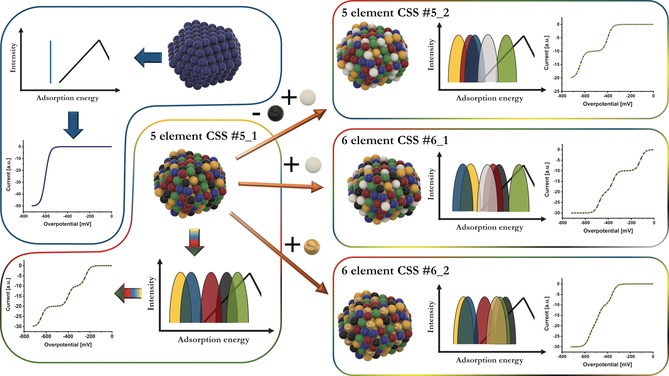
Schematic illustration of correlations between CSS nanoparticle structure, its effect on the adsorption energy distribution pattern, and the respective electrochemical response in the kinetic region. The shift in energy of the individual adsorption peaks upon replacement or addition of elements depends on the material‐inherent properties based on the complex interaction with the other elements and only few arbitrary examples of the many options are visualized.

Considering the examples in Scheme 2, replacing or adding elements induces, to date unpredictable, shifts of all individual adsorption peaks within the AEDP and theoretical or experimental evaluation of the changes is necessary. In case of the six‐element alloy CSS #6_2 (Scheme 2, bottom right), a close overlap of the three most active peaks yields a catalytic curve in which all three wave segments are hardly separated. The five‐element CSS #5_2 with replaced elements (Scheme 2, top right) shows an altered AEDP in which the third most active peak is shifted towards unfavorable binding energies and only two wave segments are visible within the considered potential regime. The CSS which is most active which has the best peak closest to the optimum is the six‐element CSS #6_1 (Scheme 2, middle). Thus, it is not yet possible to predict the effect of adding or replacing elements and both can have positive and negative effects. However, the shape of the catalytic curve in the kinetic region provides direct information about the AEDP, that is, the separation of the wave segments is a direct measure of the separation of the peaks in the AEDP and the activity of these waves is directly linked to the position of the peaks.

### Experimental Verification

For experimental evaluation of these concepts, it is favorable to have a means to measure the intrinsic activity of CSS catalysts in the absence of any matrix effects, such as mass loading, conductivity, porosity, or the influences of binders, which may mask relevant information.^15^ Therefore, we followed our previously suggested approach of potential‐assisted immobilization of NPs at etched carbon nanoelectrodes,^8^ which yields sub monolayer, well‐separated coverage of isolated NPs. Additionally, due to the increased hemispherical diffusion, the kinetic region is enlarged and the hypothesized wave segment pattern can be observed within a broader potential regime.

All NP suspensions were synthesized by combinatorial co‐sputtering^16^ into the ionic‐liquid [Bmim][(Tf)_2_N] with the advantages of high elemental purity, composition control from the physical vapor deposition technique and the absence of any surfactants or stabilizer, while having good control over the composition of the NPs with narrow size distributions. The elemental compositions for the sputtered alloys are shown in Table 1 based on EDX data, which were obtained for the sputtered thin films located next to the ionic‐liquid (IL) cavities on the substrate wafer. With the exception of Ag, all the elements are present in close to the aspired equiatomic ratio. Since all the samples are prepared in the same way utilizing the same IL, NP sizes are expected to be in the range from 2 to 5 nm with a narrow size distribution.^8^ Hence, the changes in activity curves can be solely assigned to the changes in elemental configurations and thus, the altered intrinsic properties of the active sites. Before immobilization of the CSS NPs, the blank electrode was cycled until a stable response was obtained and this current was subtracted from the current after immobilization of NPs to obtain the electrode‐corrected current, which solely represents the NP response.^17^ It is important to note that the carbon electrode is not completely inert and its much lower activity gets compensated by its much higher accessible surface area in comparison to the NPs, which have a very low loading. Hence, there will be no plateau current but a local maximum, which eventually transitions into an overshooting peak if not interrupted before by the subsequent current wave segment. However, when the 2nd current wave is following directly after the first as a result of the similar position of the respective adsorption peaks, the 2nd current increase can still be observed before the complete decline of the first wave. Thus, the qualitative peak separation within the AEDP can be extracted by analysis of the electrode‐corrected curve pattern, that is, the adsorption peak separation is directly correlated to the current wave separation.

**Table 1:** EDX analysis of sputtered thin films located next to the IL cavities for the investigated multinary alloys.

Configuration	Molar ratio in thin film [%]													
	Cr	Mn	Fe	Co	Ni	V	Ag	Cu	Mo	Nb	Ti	Zr	Ta	W
CrMnFeCoNi (I & II)^8^	21	19	22	18	20									
CrMnFeCoNi (new)^8^	20	16	20	22	22									
VCrMnFeCo	20	19	22	18		21								
CrMnFeCoNi+Ag	14	11	13	15	14		34							
CrMnFeCoNi+Cu	16	14	16	18	18			18						
CrMnFeCoNi+Mo	18	16	17	19	19				12					
CrMnFeCoNi+Nb	15	15	16	19	19					17				
TiVZrNbTa						20				19	22	22	17	
TiNbMoTaW									20	19	21		22	17
														
MnFeCoNi^8^		23	27	23	27									
CrFeCoNi^8^	25		27	23	25									
CrMnCoNi^8^	28	22		23	27									
CrMnFeNi^8^	27	24	24		25									
CrMnFeCo^8^	27	23	27	23

**Figure 1:**
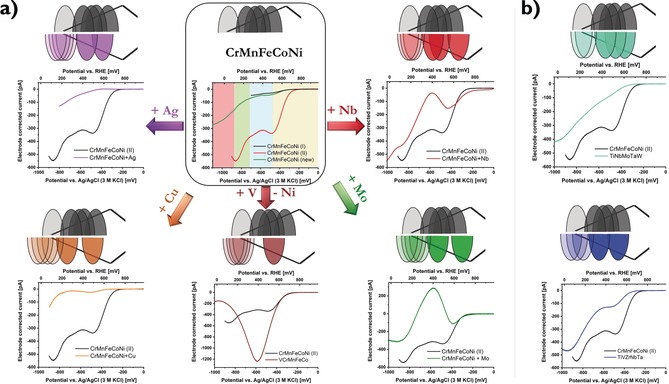
Synopsis of experimentally obtained electrode‐corrected catalytic curves of various CSSs. a) The starting CSS CrMnFeCoNi was measured 3 times using different electrodes. Even though different mass loadings yield different current intensities, the inherent wave segment pattern (different color for each wave segment) is maintained and a schematic AEDP relative to the volcano lines can be derived. The effect of addition or replacement on the experimental current wave segment pattern and the qualitative changes in the AEDP are shown. Adsorption peaks far off the optimum cause visible wave segments that are out of the considered potential region at undefined positions and are depicted with much higher transparency. b) The same comparison is highlighted for complete replacement of all five elements.

In the case of non‐noble metal alloys, it is also important to consider that some surface atoms, for example, Cr and Mn in the Cantor alloy, are probably oxidized.^18^ Hence, a challenging refinement of the theoretical model would be required, that is, how the active sites are structured in such cases. To understand its effect on catalytic properties is important for future studies. However, the distribution in adsorption peaks is derived from the presence of a CSS, which is still preserved and the general shape of the distribution pattern is presumably preserved, but reduced to those peaks, which refer to non‐oxidized elements. In Figure 1 a, three individual electrode‐corrected CrMnFeCoNi curves obtained from different electrodes are shown. Owing to statistical differences in mass loading, the current intensities are different. However, the potential regions for each wave segment are consistent as highlighted with the colored regions. Each wave segment represents one adsorption peak in the AEDP and thus, a qualitative representation of the related AEDP can be derived (black distribution pattern). With four visible current waves, four peaks can be plotted within the considered potential window and the position of the fifth peak remains unknown, but it is at even higher distance to the optimum (transparent peak at undefined position to the left).

In the next step, we investigated the effect of replacement or addition of one element on the wave segment structure and thus, a shift of the individual peaks in the qualitative AEDP. In addition to altered inter‐elemental interactions, changes in oxidation probabilities can also affect the AEDP in the case of transition‐metal CSS catalysts and knowledge about the oxidation properties of each element can help to differentiate between both effects. Upon addition of Mo, Nb, or Cu, a clear local current maximum is observed with consecutive transition to an overshooting peak at different scales because of different mass loadings. This behavior indicates splitting of the two most active current waves (since overshooting is observed for the theoretical presence of a plateau^17^) and thus, splitting of the first two adsorption peaks as well. However, instead of levelling off to zero current, the second adsorption peak causes a second current increase at higher overpotentials visible as the next current wave. Replacement of Ni by V has a similar effect, yet the second wave segment is not visible in the potential regime considered. Another possible conclusion is a very close overlap of those two waves, yielding only one combined current wave of higher intensity. Addition of Ag is not supposed to maintain a homogeneous CSS phase because of the large difference in atomic size^19^ and tends to have strong effects on the crystal structure with a change in average coordination number.^20^ It was further observed that such HEAs can have strong inhomogeneous fluctuations in the elemental distribution with local aggregations.^21^ The observed current progression also exhibits the decline of the first current wave, which is, however, followed by the second current wave before the local maximum is reached. This pattern suggests a closer position of the first two adsorption peaks within the AEDP. The same observation is obtained for the two CSSs with complete replacement of all five elements (Figure 1 b). TiNbMoTaW shows three visible wave segments, which all migrate into the following one, whereas TiVZrNbTa shows two, which are more separated.

For simplicity, all the derived qualitative AEDP only show adsorption peaks, which are shifted towards the strong‐binding side compared to optimal adsorption energy. However, only the absolute distance to the volcano maximum can be predicted, but not the direction since a shift in either direction causes the same decrease in activity. Hence, we point out that other solutions of the derived AEDP also exist, where one, a few, or all peaks are at the same distance, but on opposite sides of the volcano optimum.

### Comparison of Most Active Current Waves

**Figure 2:**
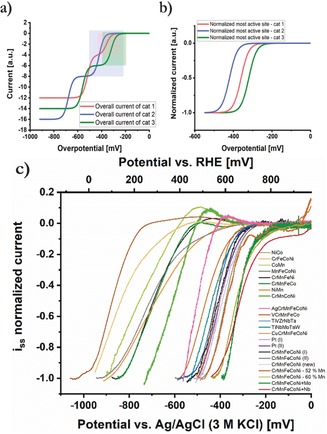
a) Schematic current‐overpotential curves of CSS catalysts forming a CSS of different configuration and mass loading. The combined current response of all the active sites within one adsorption peak of the adsorption‐energy distribution pattern is represented by one “current wave”. For a similar position of the two most active peaks regarding optimal binding energies, two “wave segments” yield the overall current response with the presence of an intermittent plateau current. b) Normalized curves of (a) respective to the plateau current (colored areas) to compare the activity of different catalysts of their respective most active adsorption peaks. c) Experimental results of ORR activity in 0.1 m KOH of selected multinary alloys immobilized at etched carbon nanoelectrodes. Normalization was performed as described in (b). The sample order is based on the required overpotential to reach −0.5 at the normalized scale. “cat”=“catalyst”.

To allow for comparison of the intrinsic activity of different catalysts, normalization of electrode‐corrected catalytic curves by the first plateau current was performed (Figure 2 a).^17^ This approach is intended to compensate for different amounts of immobilized particles. In the specific case of CSS catalysts, this method allows for comparison of the first wave segment of each catalyst (Figure 2 b) and thus, the most active adsorption peaks within the AEDP. Hence, it exactly offers the aspired information for the proposed first step. By finding the elemental configurations with promising AEDPs with suitable position of the most active peak, promising catalyst candidates can be selected and used for further composition optimization.

The experimental results of this study applied to the ORR in alkaline media are shown in Figure 2 c. For comparison, Pt NPs prepared with the same parameters serve as benchmark ORR catalyst.^8^ The activity seems to be shifted to higher overpotentials when comparing with more conventional RDE data. This is due to the utilized approach based on isolated NPs of a very low loading. The implication of an unavoidable lower signal to noise ratio is discussed in detail in the Supporting Information. As highlighted previously,^8^ the CrMnFeCoNi catalyst is significantly more active than its subsystems composed of fewer elements, which can be attributed to the presence of novel active sites within the CSS. The possibility of increasing the activity by tailoring the composition with increased Mn‐content supports the theoretical hypothesis regarding the adsorption‐energy distribution pattern.^4^ We extended the list of investigated catalysts and evaluated the effect of addition of a 6th element to the CrMnFeCoNi system. According to the model, the adsorption‐energy distribution pattern should change in a way that now six adsorption peaks are present. Owing to interaction of an additional element, the position of each peak is altered and can hardly be predicted. We also investigated the influence of a lattice distortion in the uniform elemental distribution within the CSS by addition of Ag. Its effect on intrinsic activity is a shift of the catalytic curve toward higher overpotentials implying a shift of the peak maximum in the adsorption energy diagram away from the optimal position. The same effect, but less pronounced, is observed for the addition of Cu instead of Ag. Copper is the next in number element in the first transition row of the periodic table, which indicates the preservation of the CSS phase as a result of the similar size. However, addition of Nb or Mo clearly shows an improvement in intrinsic activity. Those two alloys reach the best performance, even slightly improved compared to the adjusted CrMnFeCoNi composition with enhanced Mn content and superior of the benchmark catalyst Pt even though in these alloys an equiatomic ratio is present. Replacing one element of the quinary system CrMnFeCoNi by reducing the atomic number of each element by one (“downshift” of atomic number) to obtain VCrMnFeCo results in a decrease in activity. We also investigated the effect of complete replacement of all five elements by testing the refractory HEAs^22^ TiVZrNbTa and TiNbMoTaW. Both show very similar intrinsic activity of the most active current wave, which is only marginally lower compared to the non‐optimized CrMnFeCoNi system.

## Conclusion

To summarize, due to the continuous coverage of a wide range of adsorption energies, CSS catalysts usually possess highly active sites. This is validated by our results, showing a big gap in intrinsic activity between the binary through quaternary subsystems, and all tested alloys comprising five or more elements. Whereas the low intrinsic activity of the binary systems is consistent with the position of their elements in volcano plots, the CSS catalysts show at least an enhanced activity arising from the broad adsorption energy distribution also partially covering more favorable energies. To reach very high activities, a good fit of the best adsorption peak maximum with the optimal binding energy as well as a high intensity of this peak corresponding to a high number of those sites is required. We showed how the presence of two current waves of similar activity indicates similar positions of the two most active adsorption peaks within the AEDP, whereas a single exponential increase is an indication of a well‐separated most active peak. Although it is to date hard to predict the position of those peaks just by the selected elements, we evaluated a selection of CSS catalysts regarding the position of the most active peak and found some very active configurations. To transfer this high activity to catalyst‐film applications where the intensity also plays an important role, optimization of the composition becomes very important. Since the position of the peak is presumably not strongly affected by changes in the molar ratio, the results obtained with equiatomic compositions provide valuable information concerning promising elemental configurations. This correlation improves the search for active CSS catalysts significantly, since experimental screening can be simplified to equiatomic samples without the need to evaluate all different compositions within each set of elements as well. Thus, conceptual understanding of the general working principles of this new complex catalyst class is provided which is applicable to any reaction. Furthermore, it particularly provides a method to assess information about the intrinsic activity of abundant CSSs for the ORR. Exploiting these possibilities, we discovered CrMnFeCoNiNb and CrMnFeCoNiMo as very promising candidates for the ORR, whose composition can be optimized to complement the suitable position of the most active peak with a high intensity and thus, transfer the high activity to up‐scaled applications.

## Conflict of interest

The authors declare no conflict of interest.

## Supporting information

As a service to our authors and readers, this journal provides supporting information supplied by the authors. Such materials are peer reviewed and may be re‐organized for online delivery, but are not copy‐edited or typeset. Technical support issues arising from supporting information (other than missing files) should be addressed to the authors.

Supplementary

## References

[1a] Y. Jiao , Y. Zheng , M. Jaroniec , S. Z. Qiao , Chem. Soc. Rev. 2015, 44, 2060–2086; 25672249 10.1039/c4cs00470a

[1b] Z. W. Seh , J. Kibsgaard , C. F. Dickens , I. Chorkendorff , J. K. Nørskov , T. F. Jaramillo , Science 2017, 355, eaad4998. 10.1126/science.aad4998 28082532

[2] A. Kulkarni , S. Siahrostami , A. Patel , J. K. Nørskov , Chem. Rev. 2018, 118, 2302–2312. 29405702 10.1021/acs.chemrev.7b00488

[3] T. Löffler , A. Savan , A. Garzón-Manjón , M. Meischein , C. Scheu , A. Ludwig , W. Schuhmann , ACS Energy Lett. 2019, 4, 1206–1214.

[4] T. A. A. Batchelor , J. K. Pedersen , S. H. Winther , I. E. Castelli , K. W. Jacobsen , J. Rossmeisl , Joule 2019, 3, 834–845.

[5] Y. Yao , Z. Huang , P. Xie , S. D. Lacey , R. J. Jacob , H. Xie , F. Chen , A. Nie , T. Pu , M. Rehwoldt , et al., Science 2018, 359, 1489–1494. 29599236 10.1126/science.aan5412

[6] H.-J. Qiu , G. Fang , Y. Wen , P. Liu , G. Xie , X. Liu , S. Sun , J. Mater. Chem. A 2019, 7, 6499–6506.

[7a] J. Pedersen , T. Batchelor , A. Bagger , J. Rossmeisl , ChemRxiv 2019, 10.26434/chemrxiv.9850997.v1;

[7b] S. Nellaiappan , N. Kumar , R. Kumar , A. Parui , K. D. Malviya , K. G. Pradeep , A. K. Singh , S. Sharma , C. S. Tiwary , K. Biswas , ChemRxiv 2019, 10.26434/chemrxiv.9777218.v1.

[8] T. Löffler , H. Meyer , A. Savan , P. Wilde , A. Garzón Manjón , Y.-T. Chen , E. Ventosa , C. Scheu , A. Ludwig , W. Schuhmann , Adv. Energy Mater. 2018, 8, 1802269.

[9] M. W. Glasscott , A. D. Pendergast , S. Goines , A. R. Bishop , A. T. Hoang , C. Renault , J. E. Dick , Nat. Commun. 2019, 10, 2650. 31201304 10.1038/s41467-019-10303-z PMC6570760

[10] G. Zhang , K. Ming , J. Kang , Q. Huang , Z. Zhang , X. Zheng , X. Bi , Electrochim. Acta 2018, 279, 19–23.

[11] Z. Jin , J. Lv , H. Jia , W. Liu , H. Li , Z. Chen , X. Lin , G. Xie , X. Liu , S. Sun , et al., Small 2019, ## 10.1002/smll.201904180. 31596058

[12] W. Dai , T. Lu , Y. Pan , J. Power Sources 2019, 430, 104–111.

[13] K. D. Jensen , J. Tymoczko , J. Rossmeisl , A. S. Bandarenka , I. Chorkendorff , M. Escudero-Escribano , I. E. L. Stephens , Angew. Chem. Int. Ed. 2018, 57, 2800–2805; 10.1002/anie.201711858 29345738

[14] K. R. Ward , N. S. Lawrence , R. Seth Hartshorne , R. G. Compton , J. Electroanal. Chem. 2012, 683, 37–42.

[15] S. Zou , M. S. Burke , M. G. Kast , J. Fan , N. Danilovic , S. W. Boettcher , Chem. Mater. 2015, 27, 8011–8020.

[16] D. König , K. Richter , A. Siegel , A.-V. Mudring , A. Ludwig , Adv. Funct. Mater. 2014, 24, 2049–2056.

[17] T. Löffler , P. Wilde , D. Öhl , Y.-T. Chen , K. Tschulik , W. Schuhmann , Faraday Discuss. 2018, 210, 317–332. 29978879 10.1039/c8fd00029h

[18a] Y. J. Li , A. Kostka , A. Savan , A. Ludwig , J. Alloys Compd. 2018, 766, 1080–1085;

[18b] K. F. Quiambao , S. J. McDonnell , D. K. Schreiber , A. Y. Gerard , K. M. Freedy , P. Lu , J. E. Saal , G. S. Frankel , J. R. Scully , Acta Mater. 2019, 164, 362–376.

[19] S. Guo , C. T. Liu , Prog. Nat. Sci. Mater. Int. 2011, 21, 433–446.

[20] D. B. Miracle , O. N. Senkov , Acta Mater. 2017, 122, 448–511.

[21] Q. Ding , Y. Zhang , X. Chen , X. Fu , D. Chen , S. Chen , L. Gu , F. Wei , H. Bei , Y. Gao , et al., Nature 2019, 574, 223–227. 31597974 10.1038/s41586-019-1617-1

[22] O. N. Senkov , D. B. Miracle , K. J. Chaput , J.-P. Couzinie , J. Mater. Res. 2018, 33, 3092–3128.

