# Supplementary material for: Design of Complex Solid‐Solution Electrocatalysts by Correlating Configuration, Adsorption Energy Distribution Patterns, and Activity Curves
Source: Angew Chem Int Ed Engl. 2020 Feb 11;59(14):5844–50. doi: 10.1002/anie.201914666 (PMC7155130; doi:10.1002/anie.201914666)
Supplement: Supplementary file 1 — Supplementary [file ANIE-59-5844-s001.pdf]

Angewandte Chemie
Eine Zeitschrift der Gesellschaft Deutscher Chemiker

Supporting Information

Design of Complex Solid-Solution Electrocatalysts by Correlating Configuration, Adsorption Energy Distribution Patterns, and Activity Curves

Tobias Löffler, Alan Savan, Hajo Meyer, Michael Meischein, Valerie Strotkötter, Alfred Ludwig, and Wolfgang Schuhmann**

anie_201914666_sm_miscellaneous_information.pdf

Supporting Information

Experimental

Experimental Section

NP Synthesis by Combinatorial Co-Sputtering (Single Targets): Combinatorial co-sputter deposition[1] from five confocal elemental sources (4-inch diameter) was performed in a UHV-base vacuum deposition system (CMS 600/400 LIN, DCA Instruments Turku, Finland). Ar was used as process gas with a purity of 99.9999% (Praxair). Elemental targets were used: Cr (MaTecK, 99.95% purity), Mn (Sindlhauser, 99.95% purity), Fe (Sindlhauser, 99.9% purity), Co (Sindlhauser, 99.99% purity), and Ni (K. J. Lesker, 99.995% purity).

NP Synthesis by Combinatorial Co-Sputtering (CrMnFeCoNi alloy target with one additional element (Ag, Nb, Cu, Mo)): Combinatorial co-sputter deposition from two confocal elemental sources (1.5-inch diameter) was performed in an AJA POLARIS-5 chamber operated with two DC-XS 1500 multiple sputter source DC power supplies. Ar was used as process gas with a purity of 99.999% (Praxair). The CrMnFeCoNi alloy target was custom-made with equiatomic composition. As additional elemental targets were used: Ag (EvoChem, 99.99% purity), Nb (EvoChem, 99.95% purity), Cu (EvoChem, 99.99% purity) and Mo (EvoChem, 99.95% purity). The sample denoted as CrMnFeCoNi (new) has also been prepared with the custom made HEA target utilizing the AJA POLARIS-5 chamber.

The IL [Bmim][$(Tf)_2N$] (purity > 99%, impurities: halides (IC) < 100 ppm, water (KF) < 71 ppm by certificate analysis) was used (Iolitec). IL was stored under Ar and was used with no further purification.

40 µL of IL was pipetted under Ar into each cavity of a stainless steel holder.[2] Utilizing covers, up to 64 cavities can be simultaneously used. The holder was cleaned by ultrasonication for 20 min in acetone and isopropanol. IL and a piece of a patterned Si/SiO_2 wafer (2 cm × 3 cm, photolithographically structured with a photoresist lift-off cross pattern for film thickness determination) were placed on the cavity holder under Ar. After transferring the holder into the sputter chamber with a minimum starting vacuum of 1.9×10^{-8} Pa, the cathodes were pre-cleaned for 5 min against their shutters. The deposition was performed at an Ar pressure of 0.66 Pa. After deposition, the holder was transferred into Ar atmosphere. The IL/NP suspensions were collected and stored under Ar. The compositions of the thin films deposited on the oxidized Si substrates, which were placed during sputter deposition in between the cavities of the sample holder, were determined using EDX (Oxford INCA XAct SiLi detector) in a scanning electron microscope (SEM, JEOL JSM5800LV). A stylus profilometer (AMBIOS XP-2 Profiler) was used to measure film thicknesses, which ranged between 430 nm and 500 nm.

Fabrication of Etched Carbon Nanoelectrodes: Quartz glass capillaries (Sutter Instruments, outer diameter 1.2 mm, inner diameter 0.9 mm) were laser pulled (Sutter Instruments P-2000) to yield two conically-shaped nanopipettes with a disc opening of a few 100 nm. The nanopipettes were filled with a carbon film by pyrolysis of a propane/ butane gas mixture (Campinggaz) heated with a torch, while the tip apex was flushed with Ar inert gas from outside via a second counter flow capillary to protect from oxidation. The electrode surface area was increased by etching of the thin glass insulation with 5:1 buffered hydrofluoric solution containing 40% hydrofluoric acid (aq.) (AnalaR NORMAPUR):40% NH4Cl (aq.) (Sigma Aldrich) by immersion of the tip apex for 3 min and consecutive immersion in pure water to remove any residues.[3]

NP immobilization: 600 µl pure IL were added to 35 µl of NP in IL suspension. Afterwards, 600 µl EtOH were added to increase the diffusion rate of NPs to enhance collisions with the electrode surface. NPs were immobilized at the etched carbon nanoelectrodes by immersion of the tip in the ionic liquid suspension for 4 min while applying a potential of -400 mV vs. Ag/AgCl (3 M KCl). A pgu-BI 100 was used as potentiostat with a miniaturized Ag/AgCl (3 M KCl) reference electrode and a Pt wire in a second compartment as the counter electrode.

Electrochemical Measurements with Etched Carbon Nanoelectrodes: The activity of the blank electrode and after immobilization of NPs was measured in 0.1 M KOH with a current range of 10 nA. Cyclic voltammograms (CVs) in the potential range of +100 mV vs. Ag/AgCl (3 M KCl) to various cathodic potentials dependent on the occurrence of a current plateau for each investigated sample were performed with a scan rate of 10 mV s^{-1}. The blank electrode was measured as long as three consecutive cycles yielded a constant current profile, which served as the blank electrode signal. After immobilization of NPs, 3 CVs with the same parameters were performed. The blank electrode signal was subtracted from the third cycle to yield the electrode corrected NP signal, which was normalized by the plateau current as described in the manuscript and in literature.[4] For each sample, a new working electrode and a new KOH solution was taken.

„Shift“ of activity curves towards higher overpotentials

The scope of this work was to experimentally assess intrinsic catalytic properties of the investigated NPs in order to gain fundamental information about the working principles of this new catalyst class. For this purpose, conventional RDE analysis possesses some drawbacks in terms of additional contributions of matrix film effects such as surface area enhancement by mass loading or porosity, influence of additives or film conductivity (**Scheme 1a**). Therefore, we aimed to obtain sub-monolayer, isolated nanoparticles on an electrode surface, where the intrinsic NP current is obtained when measuring the current at the NP normalized by the surface area of the NPs (**Scheme 1b**). This current density can be

obtained with electrode current subtraction and normalization by mass transport current as describes elsewhere in detail.[41] It is important to note that whereas this approach is capable of fulfilling the above mentioned requirements, the obtained data should not be compared with data RDE measurements (not only because of the difference between intrinsic activity and film matrix activity). The reason is that aspiring a very low NP loading in order to achieve isolated, non-interfering NPs, the NPs current is also much lower compared to a fully covered electrode of the same size. A mass loading of 1 % (the loading is likely below [5]) implies a current, which is 100 times lower at every potential compared to a fully covered electrode (**Scheme 1c**). Accordingly, a magnification of the current scale by a factor of 100 yields a 1 % coverage current curve, which overlaps with the 100 % loading curve at the previous bigger scale (**Scheme 1d**). However, the possibility to scale is limited by the background noise level, which is governed, amongst others, by the electrode size, which is the same, independent from the loading. Hence, investigation of a potential, where the NP current can be distinguished from the background noise level has to be done at the same scale and due to the much lower current, this potential is shifted with decreasing loading (**Scheme 1e**). Hence, polarization curves of Pt NPs measured at a low loading appear to be shifted towards higher overpotentials, but normalizing to the respective surface area would yield the same results.

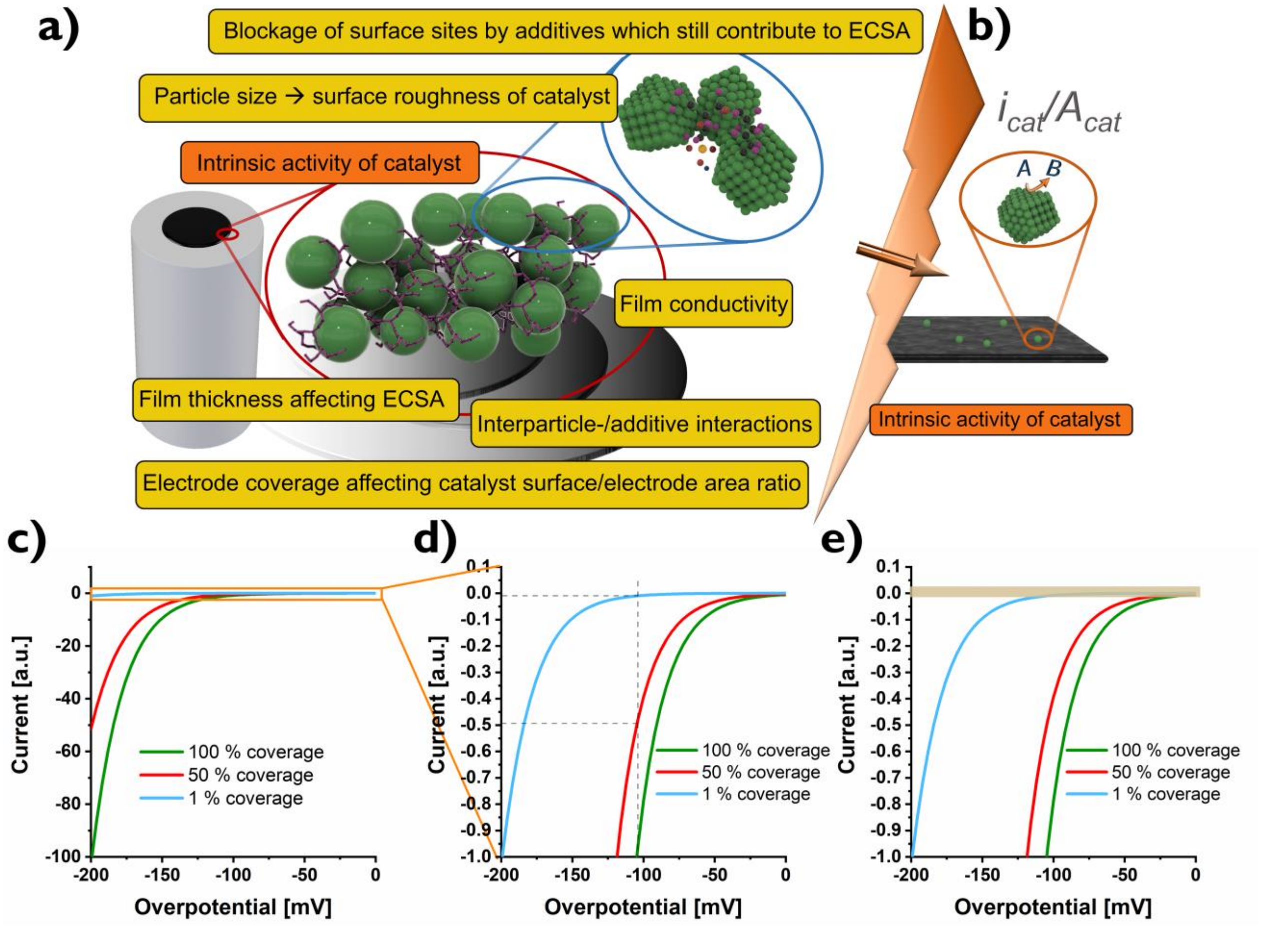

Figure 1: a) Matrix catalyst film on an RDE electrode and list of matrix factors (ECSA=electrochemical surface area), which affect the measured current response. b) Simplified case of sub-monolayer isolated NPs on an electrode surface where only the intrinsic activity is obtained when the current at the NPs is normalized by the NPs surface area. c) Respective intrinsic NPs current curves with varying electrode coverage. The factor in coverage is the same factor for the current ratio at each potential. d) Magnification of current scale by a factor of 100 with same plots as in (c) for better illustration of low current region. e) Same plot with additional background noise current, which is generally not affected by coverage. Hence, the visible catalytic current region is shifted with coverage.

References

[1] A. Ludwig, *npj Comput. Mater. (npj Computational Materials)* **2019**, *5*, 121.

[2] Y. J. Li, A. Savan, A. Kostka, H. S. Stein, A. Ludwig, *Mater. Horiz.* **2018**, *534*, 227.

[3] J. Clausmeyer, P. Wilde, T. Löffler, E. Ventosa, K. Tschulik, W. Schuhmann, *Electrochem. Commun.* **2016**, *73*, 67–70

[4] T. Löffler, P. Wilde, D. Öhl, Y.-T. Chen, K. Tschulik, W. Schuhmann, *Faraday Discuss.* **2018**, *210*, 317–332.

[5] T. Löffler, H. Meyer, A. Savan, P. Wilde, A. Garzón Manjón, Y.-T. Chen, E. Ventosa, C. Scheu, A. Ludwig, W. Schuhmann, *Adv. Energy Mater.* **2018**, *8*, 1802269.
